# In Vitro Skin Co-Delivery and Antibacterial Properties of Chitosan-Based Microparticles Containing Ascorbic Acid and Nicotinamide

**DOI:** 10.3390/life12071049

**Published:** 2022-07-14

**Authors:** Leonardo Delello Di Filippo, Jonatas Lobato Duarte, Cesar Augusto Roque-Borda, Fernando Rogério Pavan, Andreia Bagliotti Meneguin, Marlus Chorilli, Ana Melero, Antonio José Guillot, Caroline Magnani Spagnol, Marcos Antônio Correa

**Affiliations:** 1 School of Pharmaceutical Sciences, Sao Paulo State University “Julio de Mesquita Filho”, Araraquara 14800903, SP, Brazil; jl.duarte@unesp.br (J.L.D.); cesar.roque@unesp.br (C.A.R.-B.); pavanunesp@gmail.com (F.R.P.); abagliottim@hotmail.com (A.B.M.); marlus.chorilli@unesp.br (M.C.); carol.magnani@hotmail.com (C.M.S.); marcos.a.correa@unesp.br (M.A.C.); 2 Pharmaceutical Technology and Parasitology, Department of Pharmacy, University of Valencia, 46010 Valencia, Spain; ana.melero@uv.es (A.M.); antonio.guillot@uv.es (A.J.G.)

**Keywords:** polymeric microparticles, antioxidants, spray drying, skin delivery

## Abstract

Vitamins are widely found in nature, for example, in plants and fruits. Ascorbic acid and nicotinamide are examples of these compounds that have potent antioxidant properties, besides stimulating collagen production and depigmenting properties that protect the skin from premature aging. To overcome the skin barrier and reduce the instability of antioxidant compounds, alternative systems have been developed to facilitate the delivery of antioxidants, making them efficiently available to the tissue for an extended time without causing damage or toxicity. The objective of this study was to obtain chitosan biodegradable microparticles containing ascorbic acid and nicotinamide for topical delivery. The microparticles were obtained by spray drying and characterized chemically by means of scanning electron microscopy, infrared spectroscopy, X-ray diffraction, and differential exploratory calorimetry. The drugs were successfully encapsulated and the microparticles showed positive zeta potential. In vitro release assays showed a sustained release profile. The evaluation of ex vivo skin permeation and retention demonstrated low permeation and adequate retention of the compounds in the epidermis/dermis, suggesting the efficient delivery from the obtained microparticles. Antibacterial assays have shown that microparticles can inhibit the growth of microorganisms in a time- and dose-dependent manner, corroborating their use in cosmetic products for application on the skin.

## 1. Introduction

The skin is exposed daily to a myriad of environmental stresses, such as pollution and UV radiation, that can negatively impact skin health [1]. These environmental factors are known as pro-oxidant agents and are involved in the damage of cellular constituents, such as DNA, cell membrane lipids, and proteins, compromising the barrier function of the skin [2]. Oxidative damage to the skin can be caused by intrinsic and extrinsic mechanisms, which together are the primary cause of skin aging [3]. The use of antioxidants is a well-known and effective approach to treating oxidative damage in the skin [4]. The use of topical antioxidant formulations can act not only to prevent but also reverse some aspects of oxidative stress [5].

**Figure 1:**
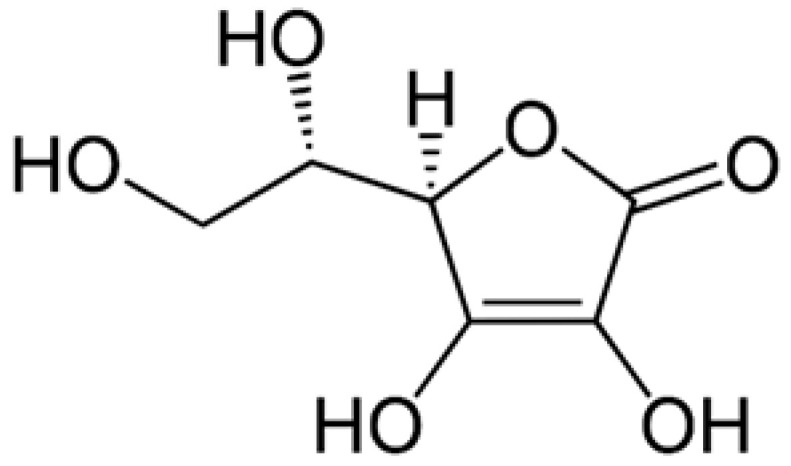
Molecular structure of ascorbic acid (AA).

The use of vitamins against oxidative damage in topical formulations is a well-known strategy [6]. Vitamin C, also known as ascorbic acid (AA), is a water-soluble vitamin that has recognized antioxidant properties, used topically in dermatological formulations to treat the damage associated with photoaging, as well as for the treatment of hyperpigmentation [7]. Due to its potent antioxidant activity, AA is widely used in topical products such as cosmetics, because it plays a key role in tissue reconstruction, protection against UV-induced photodamage, collagen synthesis, and free-radical scavenging, besides acting as a depigmenting agent. The antioxidant properties of AA are related to its molecular structure (Figure 1), capable of protecting the skin from oxidative stress by the donation of electrons to neutralize intracellular free radicals (i.e., superoxide ions, peroxide, and singlet oxygen), delaying the aging process triggered by reactive oxygen species. Despite many interesting and useful properties, the use of AA is still a challenge because it is easily degraded by changes in pH, temperature, exposure to sunlight and water, and atmospheric oxygen. Its use is usually associated with other antioxidants to decrease the oxidation of AA [7].

**Figure 2:**
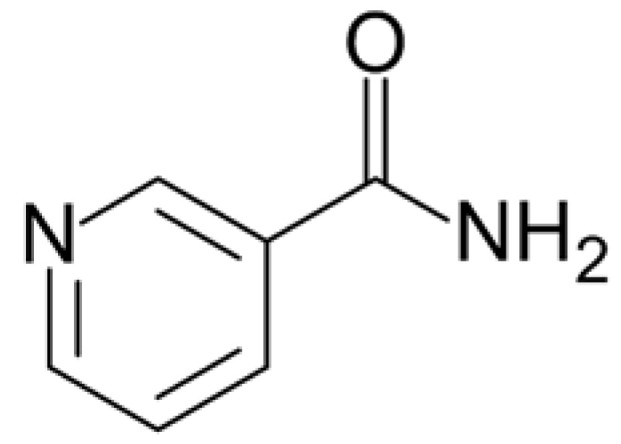
Molecular structure of nicotinamide (NIC).

Vitamin B3 (Figure 2), also known as nicotinamide, is a precursor of reduced nicotinamide coenzymes such as NADH and NADPH, which play a key role in the antioxidant cascade in the skin [8]. It can be found in cosmetic products in three forms: nicotinamide (NIC), nicotinic acid, or esters such as benzyl nicotinate and myristoyl nicotinate. NIC is the most used due to its higher tolerability (usual concentrations ranging from 2–5%) and biocompatibility, standing out for its role as a precursor in the production of NADPH and NADH, which play a role as antioxidants in various intra- and extracellular biochemical processes. Nicotinamide is also capable of decreasing inflammatory processes and sebum production, in addition to increasing collagen production, having an anti-aging and oil control effect, and being useful in the treatment of acne and rosacea. Furthermore, nicotinamide can reduce skin hyperpigmentation (as it prevents the transfer of melanin from melanocytes to keratinocytes), in addition to inhibiting protein oxidation, a spontaneous process that gives the skin a yellowish color [9].

In addition to the antioxidant intrinsic properties of NIC and AA, chitosan possesses antimicrobial properties, which encourages its use in skincare products. Therefore, a better comprehension of the antimicrobial properties of these ingredients is necessary to understand their application in cosmetics. The use of formulations containing antioxidant drugs improves the protection against oxidative damage in the skin [10,11]. However, these drugs are very unstable and may become oxidized and inactive before reaching the target tissue [12]. Given this, the use of innovative delivery systems represents a promising approach to overcoming these limitations. In addition to protecting the antioxidant drugs from premature oxidation, the use of delivery systems such as chitosan microparticles can improve biopharmaceutical properties and provide a controlled and prolonged release in the desired tissue [13]. In addition, chitosan microparticles already have well-established and easily scalable production methods.

Since there are no marketed formulations containing microencapsulated NIC and its properties in association with AA in cosmetic products are not fully known, further studies are needed to improve the exploitation of these compounds’ functions in cosmetics. This study therefore focuses on a current theme, namely, the search for new release systems for dermocosmetic molecules, aiming to develop an innovative technique that allows the effective and simple use of chitosan to improve the antioxidant and anti-aging properties of AA and NIC through encapsulation of these drugs, prolonging their time of action and increasing their bioavailability in the skin. In this context, the aim of this work was the microencapsulation of NIC and AA by chitosan microparticles (CMs) and to improve the skin delivery of these molecules. The microparticles obtained were submitted to robust physical–chemical characterization and its biological properties were evaluated using in vitro and ex vivo models.

## 2. Materials and Methods

### 2.1. Materials

Acetic acid (JT Baker, Phillipsburg, NJ, USA), ascorbic acid (Labsynth, Diadema, Brazil), trifluoroacetic acid (JT Baker, Phillipsburg, NJ, USA), anhydrous monobasic sodium phosphate (Labsynth, Diadema, Brazil),anhydrous bibasic sodium phosphate (Labsynth, Diadema, Brazil), methanol grade HPLC (JT Baker, Phillipsburg, NJ, USA), nicotinamide (Labsynth, Diadema, Brazil), medium molecular weight chitosan (190,000-310,000 Da) (Sigma-Aldrich, Barueri, Brazil) and stearylamine (Sigma-Aldrich, Barueri, Brazil).

### 2.2. Preparation of Microparticles by Spray-Drying

A 0.5% (*w*/*v*) medium molecular weight chitosan (Sigma-Aldrich) solution in acetic acid 0.1 M was prepared by mechanical stirring overnight. This dispersion was then diluted to a final concentration of 0.1% using ethanol and 1% stearylamine was added to the mixture. To this solution, a mass of AA and NIC equivalent to 15% and 30%, respectively, of the dry weight of chitosan was added.

The dispersion of chitosan (0.5% (*w*/*v*)) with AA and NIC was atomized in the BUCHI-290 Mini Spray Dryer (Buchi, France). The following parameters were programmed into the equipment: air inlet temperature, 180 °C; pump flow, 2 mL/min; atomizing airflow, 450 NL/h; aspirator efficiency, 90%; and air outlet temperature, 75 °C. 

### 2.3. Scanning Electron Microscopy

The morphology of the microparticles was determined using a high-resolution scanning electron microscope (SEM)—JEOL^®^ JSM (Jeol, Tokyo, Japan). The microparticles were deposited in small amounts on the metal surface, on double-sided carbon tape, and coated with a thin layer of gold. The sample was inserted into the equipment and, with the aid of software, the images were obtained. The average CM size was determined from SEM images using ImageJ software. At least three particles per image were considered, using five different images. The results are presented as mean size ± standard deviation.

### 2.4. Zeta Potential

The measurements were performed using the Zetasizer Nano (Malvern, Grovewood, UK). The microparticles were suspended in ultra-purified water and measurements were made in triplicate for two different samples: blank chitosan microparticles (used as standard) and chitosan microparticles containing AA and NIC.

### 2.5. Fourier-Transform Infrared (FTIR) Spectra

The spectra in the infrared region were obtained in a Shimadzu FTIR-830 spectrophotometer (Shimadzu, Tokyo, Japan). Pure KBr tablets were read as blank. The infrared spectra of pure chitosan, AA, NIC, microparticles containing AA and NIC, and the physical mixture of the ingredients used were analyzed for comparison purposes. 

### 2.6. X-ray Diffraction

X-ray diffraction was performed in an X-ray Diffractometer (XRD), Siemens, model D5000, Diffrac Plus XRD Commander (Siemens, Berlin, Germany). The measurements were performed at room temperature under Cu-Kα monochrome radiation (λ = 1.5406 Å). An open angle was used, 2θ, ranging from 0 to 90°.

### 2.7. Differential Scanning Calorimetry

DSC analyses were performed on a Mettler DSC 1 device (Mettler Toledo Switzerland) in the range of 25 °C to 300 °C, under an atmosphere of N2 (50 mL·min^−1^), with a heating ratio of 10 °C/min^−1^, using closed aluminum crucibles containing approximately 5 mg of sample. The DSC cell was calibrated before the experiments using an Indian pattern. The DSC curves of the pure AA and NIC, chitosan, and chitosan microparticles containing the drugs and their drug-polymer physical mixtures were obtained.

### 2.8. Encapsulation Efficiency

The quantification of the drugs contained in the microparticles was carried out using a mass of 5 mg of the obtained microparticles. This aliquot was solubilized in 5 mL of 0.1 M acetic acid. The mixture remained under stirring with a magnetic stirrer for 5 min and was subjected to an ultrasonic bath for another 5 min. The solution was then filtered using a 0.45 µm membrane and quantified by high-performance liquid chromatography (HPLC) (Waters, United States of America) [14]. All the measurements were made in triplicate and the results are presented as mean content ± standard deviation.

### 2.9. Release Profile

For this experiment, the microparticles were incorporated into an emulsion. The emulsion constituents initially proposed for this study (Table 1) were properly weighed on a semi-analytical scale in appropriate containers. The emulsion was prepared by a direct mixture of the aqueous phase heated to 70 °C into the oily phase at the same temperature, with manual agitation until complete homogenization [15,16]. The microparticles containing AA and NIC were solubilized in distilled water and added to the final emulsion at a concentration of 1% (*m*/*m*).

The in vitro skin release assays were performed ensuring sink conditions. The assays were conducted using modified Franz cells, with a diffusion area of 1.77 cm^2^, in Microette equipment (Hanson Research, United States of America) and cellulose membrane (Sigma-Aldrich). The receptor compartment of the modified Franz cell was filled with 7.0 mL of 0.1 M phosphate buffer (pH 5.5). For liquid samples, 300 μL was used according to the volume allowed by Franz cell attachment.

The receiving solution remained under constant agitation at 300 rpm with the use of the mini-magnetic agitators present in the Franz cells. The temperature was maintained at 37 ± 2 °C using a circulating heating bath in the jacketed cells. The evaluation of the amount of AA and NIC released from the microparticles was performed after 30 min, 1, 2, 4, 6, and 8 h. The experiments were repeated six times at each point. Quantification was performed in triplicate by HPLC according to a method previously validated [14].

### 2.10. Ex Vivo Permeation and Retention Profile

Non-scalded (in natura) swine ears were acquired from Olhos D’água Slaughterhouse (Ipuã—SP). The ears’ skins were dermatomized to obtain standardized thickness, equivalent to the epidermis and dermis (500 μm) with the aid of the Nouvag TCM300 dermatometer (Nouvag, Goldach, Switzerland).

Ex vivo skin permeation assays were performed ensuring sink conditions. They were conducted using modified Franz cells, with a diffusion area of 1.77 cm^2^, in Microette equipment (Hanson Research, Los Angeles, CA, USA). Samples were collected at 30 min, 1, 2, 4, 6, and 8 h. For this experiment the microparticles were incorporated into an emulsion system that was fully characterized (Supplementary materials).

The stratum corneum was removed by the tape stripping technique. For this, three pieces of adhesive tape (3M Scotch 720) of 1.5 cm^2^ were glued and removed from the area of the skin that was in direct contact with the receptor medium of the modified Franz cell. The adhesive tapes were dipped in methanol (4 mL) for the extraction of the drug. This suspension was then submitted to sonication for 15 min and homogenized for 1 min [17]. The supernatant was used to quantify the drugs by HPLC [14]. Then, the skin region containing the formulation–skin–receptor interface (the part of the skin where the formulation was applied, and which was in contact with the receptor medium of the modified Franz cells) was cut off. The skin fragments were immersed in methanol (4 mL) for the extraction of the drugs. This suspension was also submitted to sonication for 15 min and homogenization for 1 min. The supernatant was used to quantify the drugs by HPLC [14].

### 2.11. Antimicrobial Activity 

The antimicrobial activity was carried out using bacteria supplied by the Tuberculosis Laboratory of the Department of Biological Sciences of the FCF/UNESP. Thus, the pathogenic bacteria tested were *Escherichia coli* (ATCC 25922), *Pseudomonas aeruginosa* (ATCC 27853), *Salmonella typhimurium* (ATCC 14028), and *Staphylococcus aureus* (ATCC 25923). All bacteria were cryopreserved at glycerol 50% and stored at −80 °C.

Bacterial growth kinetics (kill time) was performed based on a previously reported protocol [18]. The microparticles were placed in a phosphate buffer pH 7.4 and DMSO (1:1) and were evaluated by serial microdilution in the concentration range of 0.25–50 mg of microparticles/mL. The absorbances were obtained at 600 nm at different times (0, 1, 6, 24, 48 h). MIC values were obtained with the same procedure but at higher concentrations (1000–4 mg/mL) according to Roque-Borda et al. [19].

Disk diffusion assays were performed with samples at a concentration of 5 mg/mL dissolved in 20% DMSO. Bacteria at 0.5 McFarland scale were seeded on Mueller Hinton Agar in contact with the diffusion disks previously immersed in each sample and incubated at 37 °C. The inhibition zone was measured using a vernier after 24 h.

## 3. Results and Discussion

**Figure 3:**
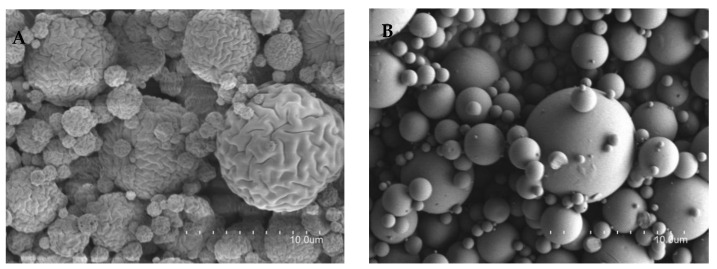
SEM of the microparticles. (**A**)—blank microparticles without stearylamine and (**B**)—AA + NIC microparticles with stearylamine; size bars: 10.0 μm.

The obtained pure chitosan microparticles presented a rough and less uniform surface (Figure 3A). The incorporation of stearylamine and the drugs in the formulations led to the formation of spherical-shaped microparticles, with a smooth and uniform surface (Figure 3B), which is a desirable attribute for uniform and controlled release systems [16]. After analysis by SEM, the microparticles presented a mean particle size of 7.53 ± 3.34 μm. 

The zeta potential of the chitosan microparticles was +39.4 ± 0.603 mV. With the addition of NIC+AA, this value decreased to +26.0 ± 5.09 mV. This can be attributed to ionizable groups of the drugs exposed to the exterior aqueous medium exhibiting a negative charge, which possibly decreased the zeta potential. The encapsulation efficiency of AA and NIC was 3.65 ± 0.82% and 6.94 ± 1.65%, respectively.

From the infrared analysis (Table 2) it was observed that drying the microparticles by atomization using a spray dryer is an efficient method of obtaining chitosan microparticles, given that the spectroscopy in the infrared region confirmed that the original components of the formulation had their molecular structure preserved, since the wavelengths of the pure substances were also found after the spray-drying process.

**Figure 4:**
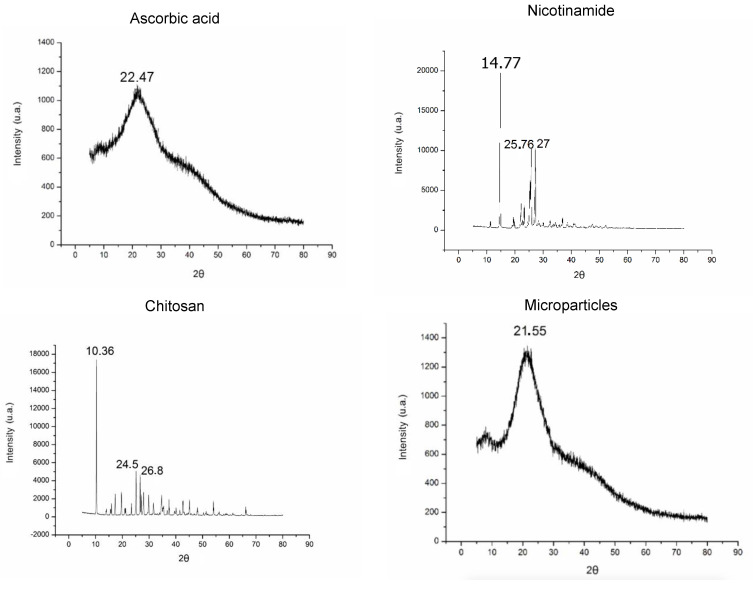
X-ray diffractograms of ingredients and AA + NIC chitosan microparticles.

The AA and NIC have crystalline characteristics which are represented by peaks in X-ray diffractograms, and the most evident peaks appear at 2θ = 10.36, 24.5, 26.8, and 14.77, 25.76, 26, respectively (Figure 4) The chitosan diffractogram did not have peaks, which is characteristic of an amorphous compound. The AA–NIC–MC diffractograms indicate that spray drying favors the formation of amorphous systems. These systems can present controlled release of drugs because their amorphous characteristic facilitates the release of drugs from the matrix. This amorphous characteristic is caused by chitosan, which generates disorganization in the crystalline structure of the drugs, allowing a solid dispersion of drugs in the polymer matrix [20].

**Figure 5:**
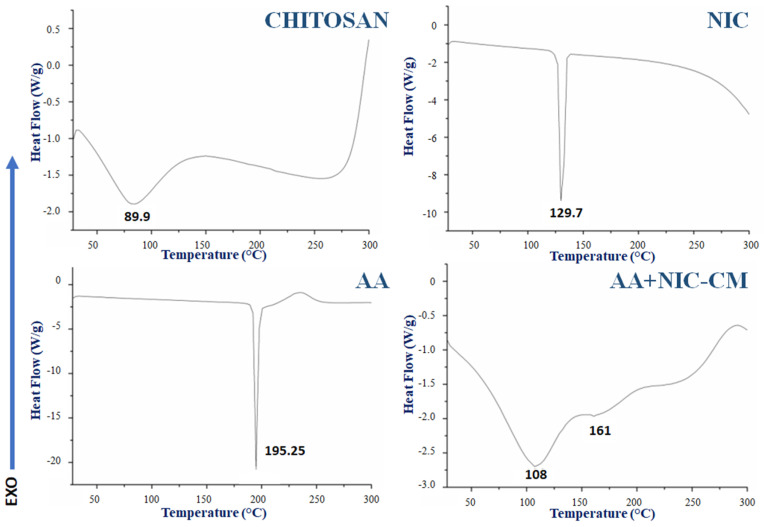
DSC–Thermograms of the endo/exothermic events produced on the chitosan microparticles (AA + NIC CMs). Chitosan powder, ascorbic acid (AA), and nicotinamide (NIC) were used as references.

The possible interactions between the drug and the polymer after the obtention of the microparticles, in addition to the thermal stability, were analyzed by differential scanning calorimetry (DSC) (Figure 5). The Chitosan–DSC curve shows that at 89 °C (endothermic, ∆ = 200.1 J/g) loss of water or solvent residues occurred and at >260 °C (exothermic) thermal decomposition took place [21]. The melting point of ascorbic acid occurs at 195.25 °C (endothermic, ∆ = 287.10 J/g) and nicotinamide 129.7 °C (∆ = 265.13 J/g); subsequent thermal events represent its decomposition [22,23,24].

In AA + NIC CMs, the loss of chitosanthermal event at 89.9 °C was due to the drying process in the microencapsulation procedure; the presence of a new band formed at 108 °C and 161 °C can be related to the glass transition temperature from chitosan, values that are usually obtained in formulations based on this polymer [25,26]. In addition, Helbling et al. (2020) indicated that obtaining a band at 161 °C could also be related to the crystallization of the drug from chitosan microparticles and the presence of various crystalline forms [27]. Ionic polymers such as chitosan can electrostatically attract bioactive substances or drugs that ionize in solution with reverse polarity to that of the polymer, forming a physical attraction that modifies the thermal response of the formulations [28]. These results demonstrate thermal stability before and after microencapsulation; likewise, cosmetic products on the market do not go through highly caloric processes, which allows the action of microencapsulation without degradation caused by heat.

**Figure 6:**
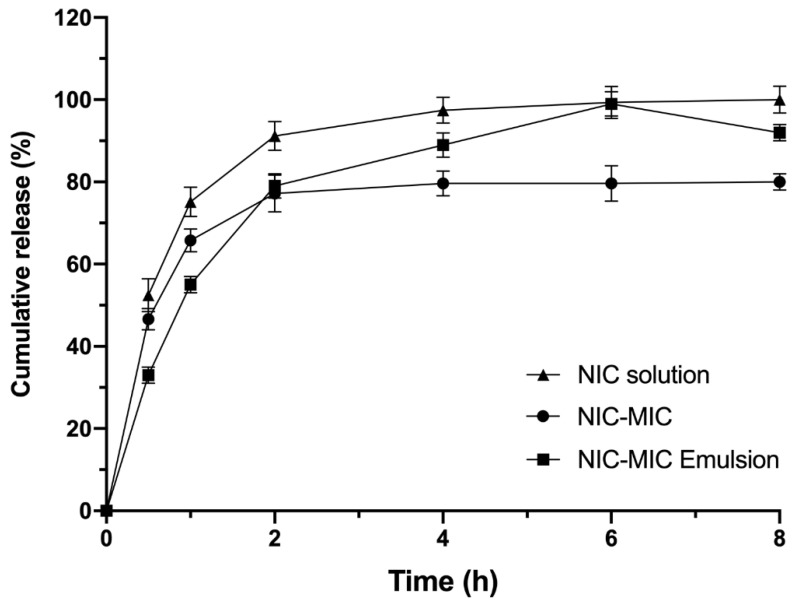
Percentage of released nicotinamide contained in microparticles at 30 min, 1, 2, 4, 6, and 8 h.

It is possible to observe that microparticles influenced the release profile of the drugs (Figure 6). For nicotinamide contained in microparticles, the release occurred more gradually, with 45% of nicotinamide being released at 30 min. It is also possible to observe that the maximum release occurred after two hours. The profile release was sustained until the end of the test, which is desirable to provide a formulation with extended antioxidant action for the skin.

For the microparticles dispersed in the emulsion, the release kinetics were slightly higher, suggesting that the emulsion, besides acting as an appropriate vehicle for the application of microparticles to the skin, also increased the drug release, that is, it favored the release of higher concentrations of antioxidants contained in the microparticles. This was probably due to its composition, with lipids, surfactants, and propylene glycol, which facilitate the erosion of the polymer matrix, favoring the diffusion of drugs. The release kinetics obtained were analyzed using various mathematical models; among them, the one that presented the highest correlation coefficient (r^2^ = 0.999) was the Weibull model. These results corroborate information found in the literature, which describes this type of release kinetics as one of the possible options for other polymeric and/or hybrid systems (containing lipids) for microparticles carrying hydrophilic drugs [29]. The diffusion profile of nicotinamide solutions was studied as experimental control, presenting faster diffusion compared to the formulations, because nicotinamide is molecularly dispersed in this vehicle. This comparison serves to evidence the development of the capacity for controlled release of the microparticles.

It was not possible to quantify ascorbic acid in this experiment, since the microparticles promote a more gradual release of the drugs, leading to only small amounts of ascorbic acid reaching the receptor solution, placing this compound outside the quantification and detection limits of the analytical method. Therefore, the quantification method was limited, since the amounts of ascorbic acid released from the microparticles could not be measured.

**Figure 7:**
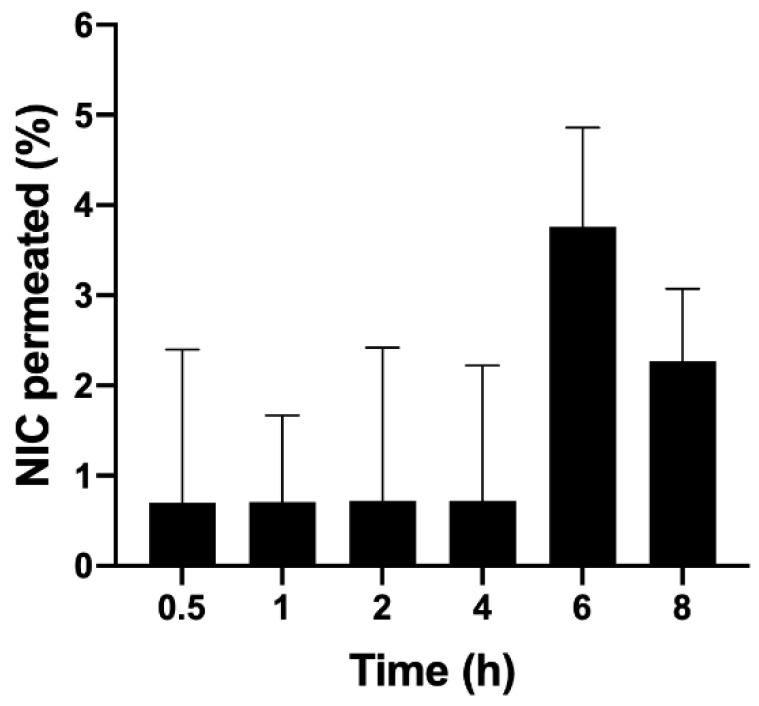
Percentage of nicotinamide permeated from the microparticles in an emulsion at 30 min, 1, 2, 4, 6, and 8 h.

In terms of ex vivo permeation and retention, the amounts of nicotinamide retained in the stratum corneum, epidermis, and dermis were detected and quantified by an analytical method validated using HPLC [14]; Figure 7 presents the results obtained. It was observed that the percentage of retention in the epidermis/dermis was higher compared to the stratum corneum, which is desirable since this tissue is where antioxidants should ideally act to minimize oxidative stress. This result suggests that CMs are able to overcome the stratum corneum, which acts as a barrier in the skin, promoting the efficient delivery of antioxidants to the epidermis/dermis.

**Figure 8:**
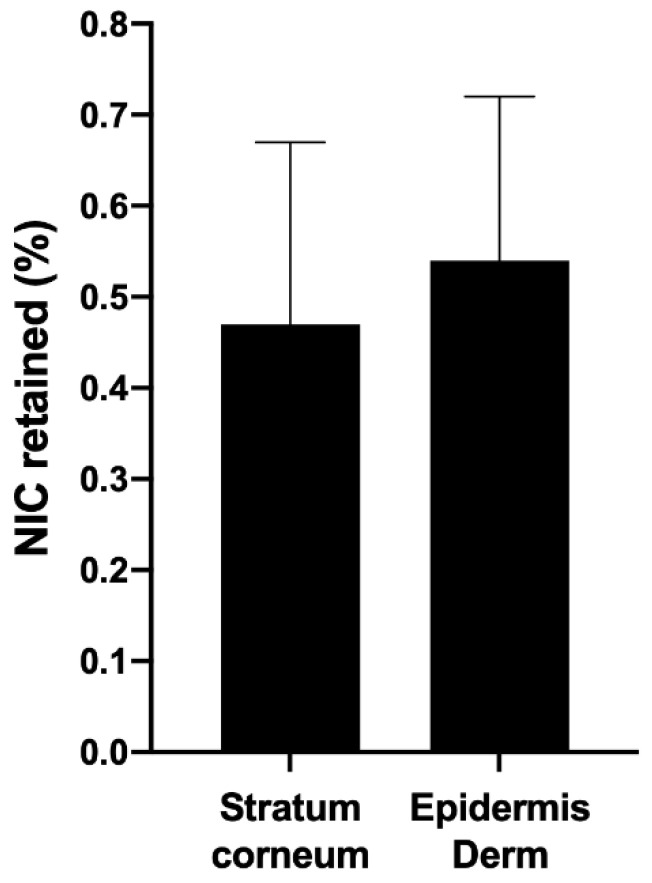
Percentage of nicotinamide retained in the stratum corneum and epidermis/dermis from the microparticles incorporated in an emulsion.

In this study, 300 mg of the emulsion containing a 1% concentration of microparticles was applied to Franz cells; the total amount of nicotinamide applied was 2082 μg. The proportion of nicotinamide retained in the epidermis and dermis from the emulsion was 0.54%. If we take 2082 μg as 100%, the 0.54% retained is equivalent to 11.24 μg, which was diluted in 4 mL of methanol, obtaining a concentration of nicotinamide in the solution equal to 2.81 μg/mL. The proportion of nicotinamide retained in the stratum corneum was 0.46%; the same reasoning was applied where a concentration of 2.39 μg/mL was obtained (Figure 8).

This result was satisfactory because, in general, it can be said that there was no significant permeation of nicotinamide, which is mandatory for a cosmetic product. The cytotoxicity profile of nicotinamide is already well known and therefore it is known that this cosmetic ingredient has good dermal tolerability, not being toxic or mutagenic, in the common amounts used in cosmetic products [30].

**Figure 9:**
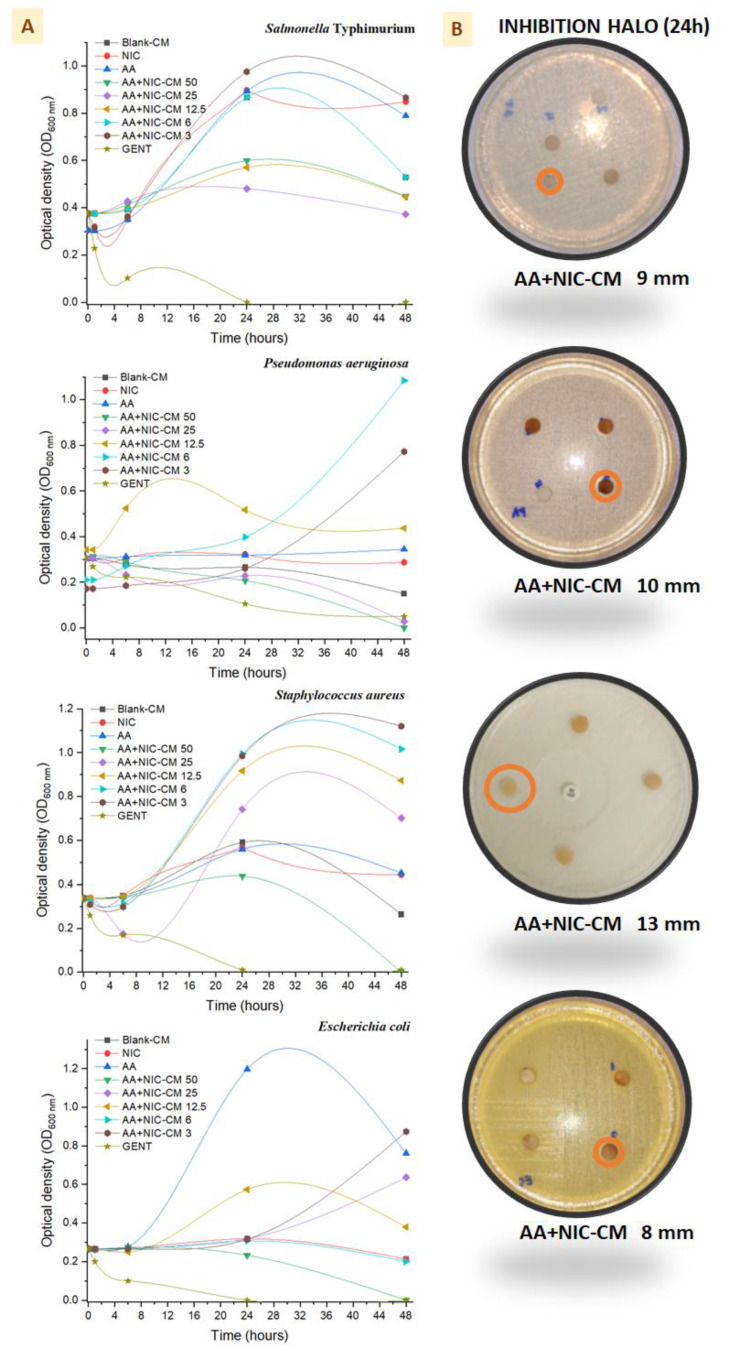
(**A**)—The kill-time curves of bacteria exposed to microparticles at different times (0, 1, 6, 24, 48 h). The concentrations used ranged from 50 to 3 mg of microparticles per mL and read at 600 nm; (**B**)—Disc diffusion test after 24 h exposure.

The minimum inhibitory concentration (MIC) values show that the original molecules did not demonstrate bactericidal activity or were above the evaluation range (>1000 mg/mL), but that they were greatly enhanced when they were encapsulated in microsystems, mainly in *S. aureus* (Table 3). The time-kill results are shown in Figure 9, where it can be seen that bacterial multiplication for all bacterial strains occurred at 0, 1, 6, 24, and 48 h. Using 3 mg/mL of microparticles (AA + NIC CMs) during the first hours (release time shown in the previous section), the growth line was controlled. However, the low stability of the drugs in aqueous media did not lead to greater consistency in antibacterial activity after 6 h, favoring the bacterial growth of all bacterial strains [31]. In addition, a higher concentration of microparticles (50 mg/mL, AA + NIC CM 50), as previously described, helped to maintain the capacity for inhibition of bacterial replication for longer, especially when chitosan was used in the formulation [32,33]. Bacterial death was not visualized at 24 h; however, at 48 h greater activity was observed with AA + NIC CM 50 for *E. coli*, *S. aureus*, and *P. aeruginosa*, and there was slight bacterial replication of *S. Typhimurium*. The microparticle results for AA + NIC CM 50 were confirmed by means of a diffusion disk assay, which enabled visualization of the presence of halos between 8 and 10 mm, mainly in *S. aureus*, which clearly showed a larger halo (13 mm) compared to the other bacteria. *S. aureus* is an extremely problematic pathogen, therefore formulations that inhibit the growth of this bacterial population are important adjuvants in cases of lesions involving the skin [34]. It was also possible to observe that the size of the halo for these bacteria during the first 12 h was 3 mm higher (data not shown) than the halo obtained at 24 h, which allows us to suggest that these microparticles would need to be supplied before the end of this period. It was also possible to observe that the size of the halo during the first 12 h for these bacteria was 3 mm higher (data not shown) than the halo obtained at 24 h, which allows us to infer that these microparticles would need to be supplied again in the same concentration before the end of this period in order to avoid population increase and possible selective pressure [35].

Curiously, the results for NIC imply that there was no alteration of replication or cell death in *P. aeruginosa*, since the absorbance was not affected when using a concentration of 50 mg/mL, which corroborates the results of time/pH stability shown in the previous section. However, in the presence of NIC, *S. typhimurium* has a higher replication curve compared to other bacteria. This bacterium probably uses NIC as a substrate or nutrient source, since some strains of *S. Typhimurium* showed this behavior [36]. In the same way, previous research has demonstrated the antimicrobial activity of AA against *S. Typhimurium* [37]. Furthermore, as mentioned above, AA’s low stability causes the molecule to degrade at pH 7.4, leading to reduced activity. This leads to a lower concentration of AA over a shorter time, and consequently the multiplication of any bacteria that were not already eradicated. These results indicate the period over which the microparticles can offer skin protection and prevent the proliferation of these pathogens, acting as a protective adjuvant in skin products.

## 4. Conclusions

Using the spray-drying technique and an ethanolic dispersion of chitosan and two natural antioxidants, it was possible to efficiently obtain polymeric microparticles with adequate physico-chemical properties. The addition of the drugs favored the formation of microparticles with a smooth and uniform surface (7 µm mean diameter). Infrared spectroscopy and X-ray diffraction analyses confirmed that the drugs were homogeneously dispersed in the polymer matrix. The microparticles showed in vitro sustained release in a skin model, as well as minimum permeation and higher epidermis/dermis retention. The emulsion increased the release of nicotinamide from microparticles. Microparticles containing AA and NIC showed antibacterial activity, suggesting they may be a useful additive in cosmetic products. Together, these results describe a technology based on a natural polymer for use on cosmetic products for the skin that is capable of facilitating the topical release of antioxidant drugs and also displays antibacterial activity.

## Figures and Tables

**Table 1:** Emulsion composition.

INCI Name	%
Cetearyl Alcohol	2.0
Ceteareth—20	4.0
Ethylhexyl Stearate	1.5
Glyceryl Stearate	1.0
Propyleneglycol	3.0
Dissodium EDTA	0.05
Methylparaben	0.18
Propylparaben	0.02
Sodium Polyacrylate (Rapithix A-100)	1.5
Water	q.s.p. 100%

**Table 2:** Characteristic bands of chitosan, stearylamine, ascorbic acid, nicotinamide, the physical mixing of components, and microparticles studied.

Chitosan	AA	NIC	Stearylamine	Physical Mixture	Microparticles
-	3524	-	-	3526	-
3365	-	3362	-	-	3351
-	-	-	3333	3318	-
-	2915	-	-	2914	2922
-	-	-	2850	2850	2850
-	1752	-	-	1752	-
-	1673	-	-	1673	-
-	1654	1652	-	-	1656
1562	-	-	-	-	1559
-	-	-	1470	1474	-
-	1100	-	-	1107	-
-	1070	-	-	1066	1068
-	-	-	-	-	-
-	1028	-	-	1028	-
-	989	-	-	984	-

**Table 3:** Minimum inhibitory concentrations (MIC) of the biomolecules and microparticles.

Sample	MIC (mg/mL)			
	ST	PA	EC	SA
Blank-CM	>500	>500	>500	>500
NIC	N/A	N/A	N/A	N/A
AA	N/A	N/A	N/A	N/A
AA + NIC CM	125	500	125	25
Gentamicin	0.002	0.004	0.008	0.004

ST = *Salmonella typhimurium*, PA = *Pseudomonas aeruginosa*, EC = *Escherichia coli*, SA = *Staphylococcus aureus*.

## Data Availability

In contact with the corresponding authors any file can be required, they are not hosted in public domain.

## Supplementary Materials

The following supporting information can be downloaded at: https://www.mdpi.com/article/10.3390/life12071049/s1.
