# Supplementary material for: In Vitro Skin Co-Delivery and Antibacterial Properties of Chitosan-Based Microparticles Containing Ascorbic Acid and Nicotinamide
Source: Life (Basel). 2022 Jul 14;12(7):1049. doi: 10.3390/life12071049 (PMC9319839; doi:10.3390/life12071049)
Supplement: Supplementary file 1 [file life-12-01049-s001.zip › life-1772144-supplementary.pdf]

In vitro skin co-delivery and antibacterial properties of chitosan-based microparticles containing ascorbic acid and nicotinamide

Leonardo Delello Di Filippo [1*], Jonatas Lobato Duarte [1] and Cesar Augusto Roque Borba[1,], Fernando Rogerio Pavan [1], Andreia Bagliotti Meneguin [1], Marlus Chorilli [1], Ana Melero Zaera [1], Antonio Guillot[1], Caroline Magnani Spagnol[1], Marcos Correa[1]

Emulsion characterization

Stability

The wide variety of raw materials currently present in the cosmetic market increasingly stimulates interest in the search for effective cosmetic preparations that provide for the well-being of the final consumer. It is therefore extremely necessary that the constituents of the excipient are compatible with each other and with the active substance to be incorporated and that the mixture is stable at sudden variations in temperature and agitation and presents, within the shelf life of the final formulation, the same characteristics of color, odor, pH, consistency, viscosity, and texture [3]. After emulsion handling, aliquots were separated that were subjected to different temperatures: 5 °C (refrigerator), 25 °C (room temperature), and 45°C. The stability study was conducted as recommended by the Stability Guide for Cosmetic Products Studies [4]. The organoleptic characteristics (appearance, color, odor) and physical characteristics (pH and density value), phase separation, and/or coalescence were considered, and the content was evaluated for 90 days, taking as a reference sample, considered standard, the evaluations of day 1 (24 h after the manipulation of the formula).

Centrifugation test

This was performed to evaluate the maintenance of the stability of the base formulations, regarding phase separation and coalescence, as recommended by the Cosmetic Products Stability Guide of the National Health Surveillance Agency (ANVISA). Centrifugation occurred at 980, 1800, and 3,000 rpm for 15 min with 5g of sample 24h after manipulation [4]

Macroscopic evaluation

The formulations were evaluated for changes in color, appearance, and odor, in addition to the observation of possible separation of phases and/or coalescence, taking as reference the evaluations of day 1 (24 h after manipulation).

Determination of pH

pH was determined in a pH meter after dilution of samples at 10%, according to the proposed protocol (BRASIL, 2004). The pH values were determined in triplicate, for 90 days (accelerated stability), in the samples submitted to temperatures -5oC, 25oC, and 45°C [4].

Determination of density

The determination of the relative density of the formulations was performed by pycnometer, as described in the Brazilian Pharmacopoeia (2010) during physical stability studies. The relative density value was obtained after the calculation shown in Equation 1.

Equation 1. Relative density

$$D_r = \frac{M_c - M_v}{M_a - M_v}$$

in which:

Dr= relative density

Mc= Pycnometer mass with emulsion

Mv= Empty pycnometer mass

Ma= Pycnometer mass with water

Rheological characterization

The rheological characterization was performed 24 h after emulsion manipulation, for samples packed at temperatures of 5 °C, 25 °C, and 45 °C (BRASIL, 2004). The proposed formulations were analyzed in a Haake rheoscale Rheostress RS-1 model, using a cone-plate sensor (C35/2°Ti), and the data were analyzed by the Origin 7.0 Software. The flow curve was performed with a shear rate of 0-100 s-1 for a period of 120 s for ascending curve and 120 s for the descending curve with a shear rate of 100-0 s-1. The flow property of the samples, thixotropy, and viscosity were determined, obtaining data every second. During the tests, the temperature was maintained at 25°C

Results and discussion

Emulsion development

The formulation obtained was white and consistent, smooth with a uniform texture (Figure 1). After the process of handling the base emulsion, microparticles equal to 1% by mass were added to the amount of emulsion used. After being properly incorporated, it was followed with subsequent studies.

Figure 1. Base emulsion with microparticles incorporated on the day of manipulation.

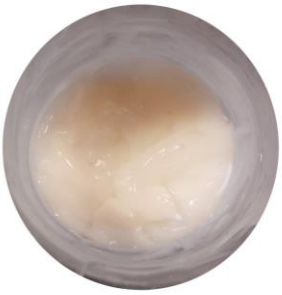

Centrifugation test

It was possible to verify that, even with the highest rotation, the formulation did not suffer any macroscopic alteration or phase separation (Figure 2), thus characterizing it as an emulsified system suitable for use.

Figure 2. Centrifugation at 980 rpm; B = Centrifuge at 1800 rpm; C = Centrifuge at 3000 rpm.

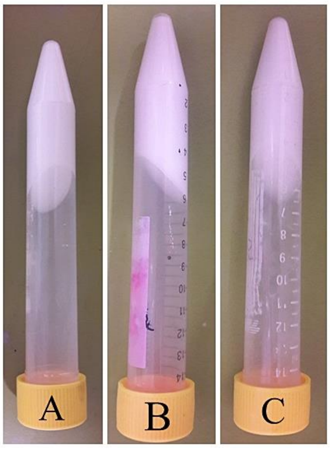

Macroscopic evaluation

Through the following records, it is possible to observe that the emulsion, compared to what was obtained shortly after manipulation, did not suffer any

apparent macroscopic changes, such as phase separation and/or coalescence, However, it is possible to observe a slight change in the color of the formulation; this can be attributed to the degradation of ascorbic acid, which generates metabolites that are present as characteristic of the yellowish coloration (Fig 3)

Figure 3. A = Sample stored at room temperature (25 °C), 90 days after manipulation, B = Sample stored at 45ºC, 90 days after manipulation; C = Sample packed at 5ºC, 90 days after manipulation.

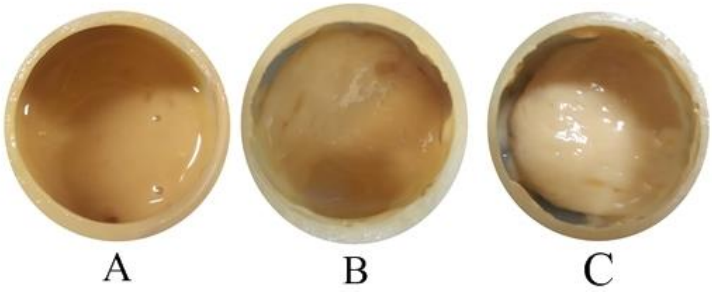

Determination of pH

On the day of manipulation, the emulsion had its pH measured, presenting a value equal to 4.05. During the stability study, the emulsion had its pH measured, in triplicate, for each of the three samples. The results after 90 days are found in Table 01.

Table 01. PH values for stability testing on days 30, 60, and 90 after manipulation.

Days after manipulation	25 °C	45 ºC	5ºC
30	4	4.1	4.04
60	4.08	4.09	4.1
90	4.1	4.03	4.05
Average pH	4,06 ± 0.05	4,07 ± 0.07	4,06 ± 0.03

Thus, it is possible to observe that, even after 90 days, the pH of the emulsions containing the microparticles, packed under different conditions, did not present significant variation.

Density determination

The results for 30, 60, and 90 days after manipulation are described in Table 02:

Table 02. Relative density results for samples stored at different temperatures, 30, 60, and 90 days after manipulation.

Days after manipulation	25 °C	45 °C	5 °C
30	0.875	0.883	0.884
60	0.852	0,827	0.875
90	0.851	0,854	0.846
Average density	0.859 ± 0.01	0.855 ± 0.02	0.858 ± 0.01

It is possible to observe that there was no significant change in the emulsion density value after 90 days of testing.

Table 03. Minimum apparent viscosity values in different temperatures.

Temperature (ºC)	Minimum apparent viscosity (Pa.s)	Hysteresis area (Dinas/cm^2.s)
25	0,7565	1305
45	0,3998	690
5	1,942	3921

These results allowed evaluation, from the flow tests, of the rheological properties of the proposed formulations. Next are the rheograms, which characterize the rheological behavior of the proposed emulsion, packed at different temperatures.

Figure 4. Flow scan of formulations stored at 25 °C, 45 °C, and 5 °C.

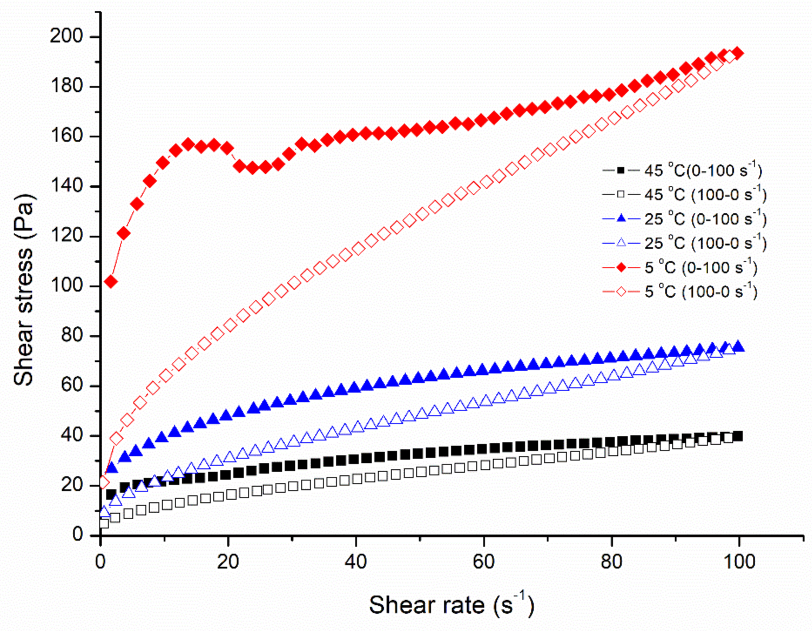

Figure 5. Viscosity versus the shear rate of formulations stored at 25 °C, 45 °C, and 5 °C

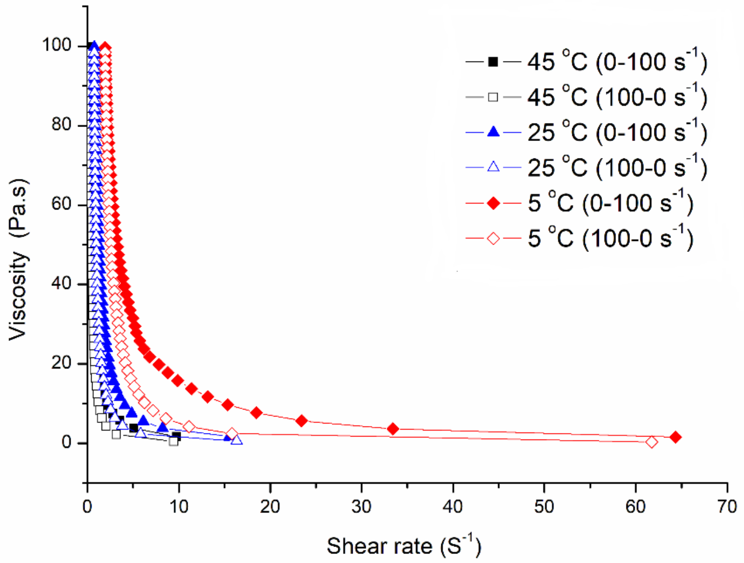

The rheograms of the emulsified system (Figures 4 and 5) show that the proposed formulation behaves like a non-Newtonian system because, unlike Newtonians, it presents a nonlinear relationship between shear stress and shear rate (MILAN et al., 2007). In addition, this system presents the characteristics of pseudoplastic fluids. This behavior is due to the alignment of colloidal droplets existing in the system, in the direction of the flow. As the tension or shear rate increases and this orientational preference is accentuated, the internal resistance of the system is reduced, promoting a consequent reduction in viscosity.

Formulations that exhibit pseudoplastic behavior produce a protective film that allows covering the skin surface, promoting better protection. Materials classified as Newtonians do not behave in this way, since spread on the skin, they tend to drain very fast. Typical pseudoplastic preparations contain interlaced or aggregated molecules, which break under shear stress and align under the direction of the flow, promoting a slip facilitated by increasing the shear speed and, consequently, reducing viscosity [5]. Viscosity recovery is usually reversible and may be dependent on time, the subsequent reconstruction of which may be due to the Brownian movement [6].

In addition, the proposed formulation also presented a thixotropic character, because it presented an area between the ascending and descending curves of the rheogram (hysteresis area)l that is, after having suffered increasing shear (0-100 s-1), with the gradual reduction in this shear until it ceased, a total recovery of the structure was not observed. This gives the formulation a certain degree of elasticity, enhancing the ability to recover its initial structure after being submitted to external forces [7].

The thixotropic character occurs due to the gradual destruction of the structure constructed by the droplets of the dispersed phase, whose interaction forces do not resist the action imposed by shear. The phenomenon is considered reversible because, after the removal of external effort (shear), broken bonds are reconstituted, reorganizing the internal structure [8].

Furthermore, it is important to highlight that the thixotropic product tends to have a longer shelf-life because during storage it presents constant viscosity, which makes it difficult to separate the constituents of the formulation. In addition to this advantage, obtaining topical formulations with thixotropic character is highly desired, because they deform during the application; that is, they become more fluid, facilitating the spread and the recovery of the initial viscosity at the moment the application is closed, which prevents the product from draining and remaining where it was applied, covering the skin. On the other hand, it is interesting to obtain a moderate value of thixotropy so that the product does not drain off the skin after application due to a very slow recovery of its structure, and a not very low value, as this can lead to low spreadability of the product, not allowing a uniform distribution on the skin [8,9]

Figure 5 shows that the formulation, although stored under different temperature conditions, presents very similar behavior for low shear rate values (up to 7 Pa.s), in all three cases. For values above 10 Pa.s, the emulsion packed in a refrigerator showed higher viscosity. This can be attributed to the reduction in entropy of the system, caused by the lowering of the temperature.

Further tests were then carried out. The frequency scanning assay, presented in Figure 6, is used in the characterization of products that present viscoelastic behavior. According to this result, the proposed emulsion can be characterized as viscoelastic, because, in the three different temperature conditions, it presented an elastic modulus (G΄) superior to the viscous modulus (G΄΄). This is an advantageous feature for the proposed purposes for this emulsion, demonstrating the greater stability of this product.

Figure 6. Frequency scan

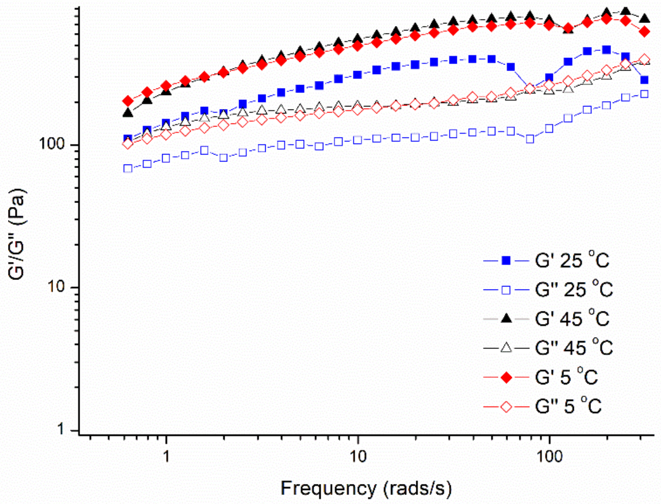

Figure 7. Fluency and relaxation test.

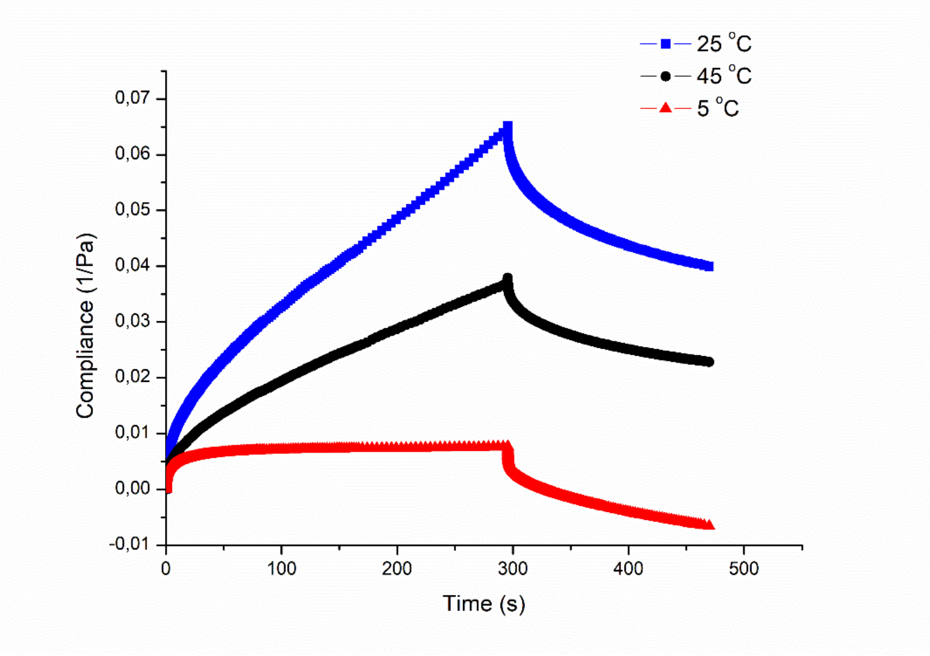

From the observed response profile (Figure 7), it is possible to verify that the formulation is viscoelastic, as had already been verified in the frequency scanning assay. Table 04 shows the complicity values obtained in this assay, a variable that demonstrates how compliant a sample is, i.e.: the higher the compliance, the easier it will be to deform by an applied stress and lower its viscosity [10].

Table 04. The maximum value of compliance and recovery for formulations stored at different temperatures.

	Temperature		
	25 ºC	45 ºC	-5 ºC
Maximum compliance (1/Pa)	0,064	0,037	0,0074
Recovery (1/Pa)	0,025	0,015	0,0008

In addition, the emulsion at room temperature showed greater recovery of its structure after ceasing the application of the tension. This is an important feature because it shows that the product spreads well on the skin at the time of

application; however, it can return to the initial state when the voltage is removed. The viscoelastic behavior of the fluid means that it has characteristics like those of a liquid and a solid concomitantly [11]
